# Prognostic significance of residual functional SYNTAX score II in acute myocardial infarction

**DOI:** 10.1371/journal.pone.0340784

**Published:** 2026-01-23

**Authors:** Xinjun Lin, Zhendong Cheng, Wei Ji, Zhibin Mei, Yaoguo Wang, Chaoxiang Xu

**Affiliations:** 1 Department of Cardiology, the Second Affiliated Hospital of Fujian Medical University, Quanzhou, Fujian, China; 2 The Second Affiliated Hospital of Fujian Medical University, Quanzhou, Fujian, China; Azienda Ospedaliero Universitaria Careggi, ITALY

## Abstract

**Background:**

The residual functional SYNTAX (Synergy Between Percutaneous Coronary Intervention with Taxus and Cardiac Surgery) score (rFSS) predicts patient prognosis post-percutaneous coronary intervention (PCI). We aimed to integrate rFSS with clinical risk factors (rFSS-II) to comprehensively assess patient prognosis, guided by the quantitative flow ratio (QFR), and compare its predictive efficacy with other scoring systems.

**Methods:**

We enrolled 175 acute myocardial infarction (AMI) patients undergoing post-PCI physiological measurements. Three distinct models were established based on different calculation methods of rFSS-II, and their respective abilities to predict major adverse cardiac events (MACE) were evaluated. Patients were categorized into high rFSS-II (n = 70) and low rFSS-II (n = 105) groups. The MACE incidence was compared between these groups over a 3-year follow-up period, using multivariable Cox regression analysis to identify MACE predictors.

**Results:**

High rFSS-II patients had more MACE than low rFSS-II patients (55.7% vs. 18.1%; p < 0.001). The rFSS-II model had higher accuracy than other traditional risk scores, except for rSS-II. In a multivariable-adjusted model, rFSS-II emerged as an independent predictor of MACE (adjusted hazard ratio: 1.08 per 1 unit increase, 95% confidence interval [CI]: 1.05–1.11, p < 0.001).

**Conclusions:**

QFR-guided rFSS-II proved to have good predictive value among AMI patients 3 years post-PCI. Calculating the total score of all vascular segments with QFR ≤ 0.80 may provide enhanced predictive capability.

## Introduction

Percutaneous coronary intervention (PCI) is a crucial treatment for acute myocardial infarction (AMI) [1]. Since 2012, the residual SYNTAX (SYNergy Between Percutaneous Coronary Intervention with TAXus and Cardiac Surgery) score (rSS) has been used to quantify the degree of residual coronary artery stenosis post-PCI, and it correlates with poorer in-hospital and long-term outcomes [2]. However, focusing solely on anatomical factors may overlook individual variabilities induced by clinical factors, potentially leading to inaccurate risk stratification. Therefore, combining clinical risk factors from SYNTAX score II (SS-II) with rSS to calculate the residual SYNTAX score II (rSS-II) could provide enhanced prognostic value. These variables include chronic obstructive pulmonary disease (COPD), peripheral arterial disease (PAD), estimated glomerular filtration rate (eGFR), left ventricular ejection fraction (LVEF), unprotected left main (LM) disease, age, and sex [3]. A previous study demonstrated a positive correlation between rSS-II and the risk of death and readmission post-PCI [4].

Discrepancies have been identified between functional significance determined using fractional flow reserve (FFR) and the severity of anatomical lesions [5,6]. To address this, the FFR-guided residual functional SYNTAX score (rFSS), which integrates anatomical and functional information, has emerged in recent years. Studies have demonstrated its superiority to rSS in evaluating poor patient prognosis post-PCI [7,8]. However, concerns regarding increased costs, prolonged operation times, and potential complications associated with pressure wire equipment have limited its widespread adoption [9].

Quantitative flow ratio (QFR) has emerged as a novel, faster, and more cost-effective method for deriving FFR without requiring a pressure wire [10,11]. Previous studies have validated FFR as the gold standard for diagnosing hemodynamically significant coronary stenosis [11–13]. Tang et al. combined QFR with rSS to study coronary arteries with QFR ≤ 0.80 in vessels larger than 2 mm in diameter. They found that this combination had improved discriminative power for predicting clinical outcomes compared with anatomical rSS alone [14].

However, it remains unclear whether quantitative score (rFSS-II), based on QFR-guided rFSS and clinical factors, is associated with long-term prognoses following revascularization in patients with AMI. In this study, we evaluated the relationship between rFSS-II and clinical outcomes post-PCI in patients with AMI, and compared it with the predictive value of other traditional scores.

## Methods

### Study population

**Fig 1:**
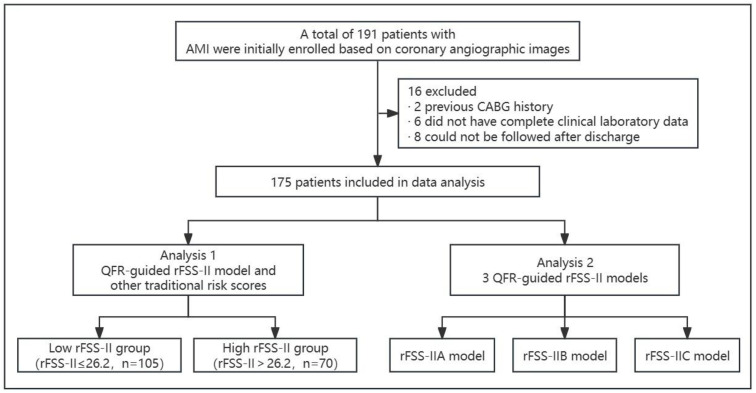
Study Flowchart: AMI, acute myocardial infarction; CABG, coronary artery bypass grafting; QFR, quantitative flow ratio; rFSS-II, residual functional SYNTAX score II.

This single-center, observational cohort study involved patients with AMI who underwent PCI at the Second Affiliated Hospital of Fujian Medical University (Fujian, China) between July 2019 and December 2020. To ensure adequate image clarity for QFR analysis using Digital Subtraction Angiography (DSA), we have established stringent criteria for the selection of images. Only high-frame-rate DSA images, with a minimum of 15 frames per second, were deemed suitable for inclusion. Furthermore, to ensure uniformity and reduce variability in image quality, we have included only those patients imaged with Philips AlluraXper FD 20 DSA equipment. Our preliminary cohort comprises 191 patients diagnosed with AMI and were diagnosed according to the 2018 Fourth Universal Definition of Myocardial Infarction [15]. Next, we excluded patients with a history of coronary artery bypass grafting (CABG), those lacking complete clinical laboratory data, and those who could not be followed up after discharge. The primary PCI strategy and decisions regarding stent placement were made at the operator’s discretion. Aspirin was administered at a daily dose of 100 mg indefinitely, with dual anti-platelet therapy of aspirin and a P2Y12 receptor inhibitor recommended for 6–12 months. In total, 175 patients were available for examination at the 3-year follow-up (Fig 1). This study followed the principle of anonymisation, and all data had personally identifiable information removed before analysis. This study was approved by the Ethics Committee of the Second Affiliated Hospital of Fujian Medical University, and the ethics committee waived the signing of informed consent. Data were accessed for research purposes on 01/04/2024.

### Definitions of the residual functional SYNTAX score II

QFR values were determined using the QFR measurement system 2.0 (AngioPlus Galley, Pulse Medical Imaging Technology, Shanghai, China). rSS was calculated by two experienced interventional cardiologists using the basic SS algorithm based on post-PCI residual coronary angiography. In scenarios where a divergence in the computational outcomes is identified between two experts, an additional expert is engaged to conduct an objective assessment, providing an adjudicative evaluation to resolve the discrepancy. The basic SS algorithm assigns scores to coronary lesions with >50% diameter stenosis in vessels larger than 1.5 mm in diameter [16].

**Fig 2:**
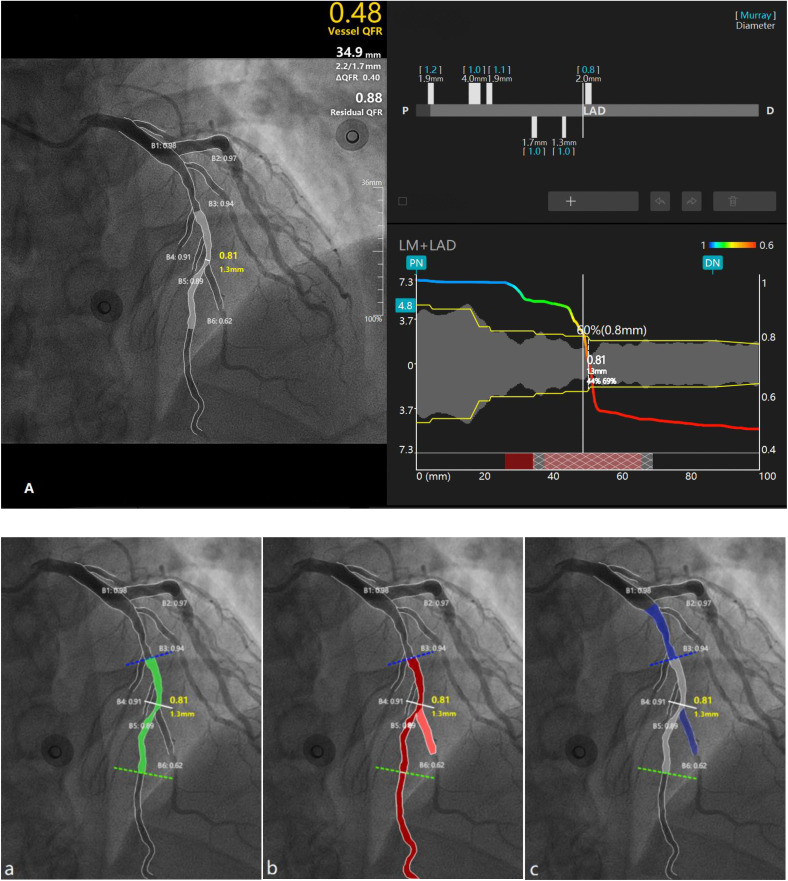
The figure illustrates different models with distinct criteria for selecting vessels. The vessel below the white solid line represents the functional ischemic part of the vessel (QFR ≤ 0.80), while anatomically significant stenosis (more than 50% stenosis) is depicted in the middle segment (grey vessel) in (A). For example, according to the basic SS algorithm, the LAD vessel is divided into proximal, middle, and distal segments marked by the blue solid line and green dashed line. In Model A (a), only segments containing anatomically significant stenosis were calculated; hence, only the middle segment of the vessel (green segment) was included, while the distal segment and branch B6 were excluded due to the absence of stenosis. All functional ischemic vascular segments were selected in Model B (b), represented by the red segments. In Model C (c), the proximal vascular segment was selected due to an anatomical stenosis (albeit less than 50% stenosis) and branch B6 was selected due to functional ischemia, and the sum was depicted as the blue segment. QFR, quantitative flow ratio; LAD: left anterior descending.

There is no universally accepted definition for computing QFR-guided rFSS-II. In this study, we designed three scoring models based on different calculation methods (Fig 2). All models required vessel diameters exceeding 1.5 mm.

Model A: Calculates the total score for all vascular segments with residual vessel stenosis >50% and QFR ≤ 0.80.

Model B: Calculates the total score for all vascular segments with QFR ≤ 0.80, regardless of the presence of stenosis.

Model C: Select vessels with a total QFR ≤ 0.80 for the entire vessel. It calculates only the fraction of the vascular segment located at the proximal stenosis (regardless of the degree of stenosis). If there is no significant stenosis in the entire vessel, it calculates the fraction of the vascular segment where the virtual stent is located. Simultaneously, branch vessels with QFR ≤ 0.80 are calculated using the basic SS algorithm, and all results are summed for the total score. When comparing rFSS-II with other scores, we defaulted to using the more acceptable Model A.

rSS and rFSS were combined with clinical factors, and the corresponding scores were obtained using the SS online calculator (https://syntaxscore.org/calculator).

### Outcomes and follow-up

The primary endpoint of this study was the occurrence of major adverse cardiac events (MACEs) over 3 years. MACE includes all-cause mortality, any myocardial infarction (MI), or ischemia-driven revascularization. Patient data were obtained from national electronic medical records, and long-term follow-up was conducted via telephone, regardless of whether the records included subsequent medical visits.

### Statistical analysis

Normally distributed continuous variables were compared using Student’s t-test and are presented as mean ± standard deviation. Continuous variables with skewed distribution were compared using the Mann–Whitney U test and are presented as median. Categorical variable data were compared using the chi-square or Fisher’s exact test and are presented as n (%). The optimal rFSS-II cut-off value associated with the primary endpoint was identified using receiver operating characteristic (ROC) curve analysis and Youden’s index. The association of rFSS-II with the cumulative 3-year rate of clinical outcomes was estimated using the Kaplan–Meier method and compared by the log-rank test. ROC curve analysis was performed to evaluate the predictive rSS, rFSS, rSS-II, and rFSS-II values for MACEs. A multivariable Cox proportional hazards regression model was used to identify independent predictors of clinical events during the 3-year follow-up after PCI in patients with AMI, and adjusted hazard ratios (HR) and 95% confidence intervals (CI) were calculated. A P-value of <0.05 was considered to indicate statistical significance. All statistical analyses were performed using SPSS version 27.0 (SPSS Inc., Chicago, III, USA).

## Results

### Baseline characteristics

The 175 patients who met the inclusion criteria and completed follow-up were included in this analysis. Most patients presented with ST-elevation MI. The mean age of the patients was 61.54 ± 12.23 years, with females accounting for 19.4% of the cohort (n = 34). Patients with hypertension, diabetes, and hyperlipidemia accounted for 52.0%, 35.8%, and 46.9% of the cohort, respectively. Fewer patients had a history of previous PCI or had comorbidities, such as COPD and PAD. Patients who experienced MACEs were significantly older and had lower eGFR levels, with differences observed in all scores (rSS, rFSS, rSS-II, and rFSS-II) between the two groups (Table 1).

**Table 1:** Baseline characteristics of the patients stratifed by the primary endpoint.

Per-patient analysis	All subjects(n = 175)	MACE-free(n = 117)	MACE(n = 58)	P value
Age, years	61.54 ± 12.23	58.93 ± 10.42	66.79 ± 13.91	<.001
Female	34(19.4)	22(18.8)	12(20.7)	0.767
Hypertension	91(52.0)	59(50.4)	32(55.2)	0.554
Smoking	85(48.6)	57(48.7)	28(48.3)	0.956
Hyperlipidemia	82(46.9)	60(51.3)	22(37.9)	0.096
Diabetes	53(30.3)	32(27.4)	21(36.2)	0.230
STEMI	157(89.7)	103(88.0)	54(93.1)	0.299
Previous PCI	10(5.7)	6(5.1)	4(6.9)	0.898
COPD	5(2.9)	4(3.4)	1(1.7)	0.880
PAD	2(1.1)	0(0.0)	2(3.4)	0.109
eGFR, mL/min/1.73m2	78.95 ± 31.07	85.13 ± 27.59	66.49 ± 34.10	<.001
LVEF, %	51.27 ± 9.65	51.88 ± 9.23	50.05 ± 10.42	0.239
BMI, kg/m2	24.18 ± 3.11	24.30 ± 3.11	23.91 ± 3.12	0.437
Angiographic disease severity				0.047
1-vessel disease	50(28.6)	38(32.5)	12(20.7)	
2-vessel disease	54(30.9)	39(33.3)	15(25.9)	
3-vessel disease	71(40.6)	40(34.2)	31(53.4)	
Residual functional severity				0.062
Insignificant stenosis	77(44.0)	57(48.7)	20(34.5)	
1-vessel disease	67(38.3)	45(38.5)	22(37.9)	
2-vessel disease	29(16.6)	14(12.0)	15(25.9)	
3-vessel disease	2(1.1)	1(0.9)	1(1.7)	
Culprit vessel				0.106
LAD	83(47.4)	51(43.6)	32(55.2)	
LCX	21(12.0)	18(15.4)	3(5.2)	
RCA	71(40.6)	48(41.0)	23(39.7)	
Severity of the culprit vessel				0.576
Severe Stenosis	36(20.6)	24(20.5)	12(20.7)	
Subtotal occlusion	13(7.4)	7(6.0)	6(10.3)	
Total occlusion	126(72.0)	86(73.5)	40(69.0)	
DCB	9(5.1)	6(5.1)	3(5.2)	>.999
DES	156(90.2)	105(91.3)	51(87.9)	0.482
rSS	3(1.00,7.00)	2.00(0.50,5.50)	5.00(2.00,10.00)	<.001
rFSS	0(0.00,3.00)	0.00(0.00,2.00)	2.00(0.00,6.25)	<.001
rSS-II	24.10(18.50,33.10)	22.30(17.30,29.50)	30.35(22.88,40.55)	<.001
rFSS-II	23.80(17.60,32.10)	21.50(16.65,29.25)	30.05(21.88,38.03)	<.001

Data are presented as mean±SD, median (IQR) or n (%). MACEs, major adverse cardiac events; STEMI, ST-elevation myocardial infarction; PCI, percutaneous coronary intervention; COPD, chronic obstructive pulmonary disease; PAD, peripheral arterial disease; eGFR, estimated glomerular filtration rate; LVEF, left ventricular ejection fraction; BMI, body mass index; LAD, left anterior descending; LCX: left circumflex; RCA, right coronary artery; DCB, drug-coated balloon; DES, drug-eluting stents; rSS:residual SYNTAX score; rFSS, residual functional SYNTAX score; rSS-II, residual SYNTAX score II; rFSS-II, residual functional SYNTAX score II.

**Fig 3:**
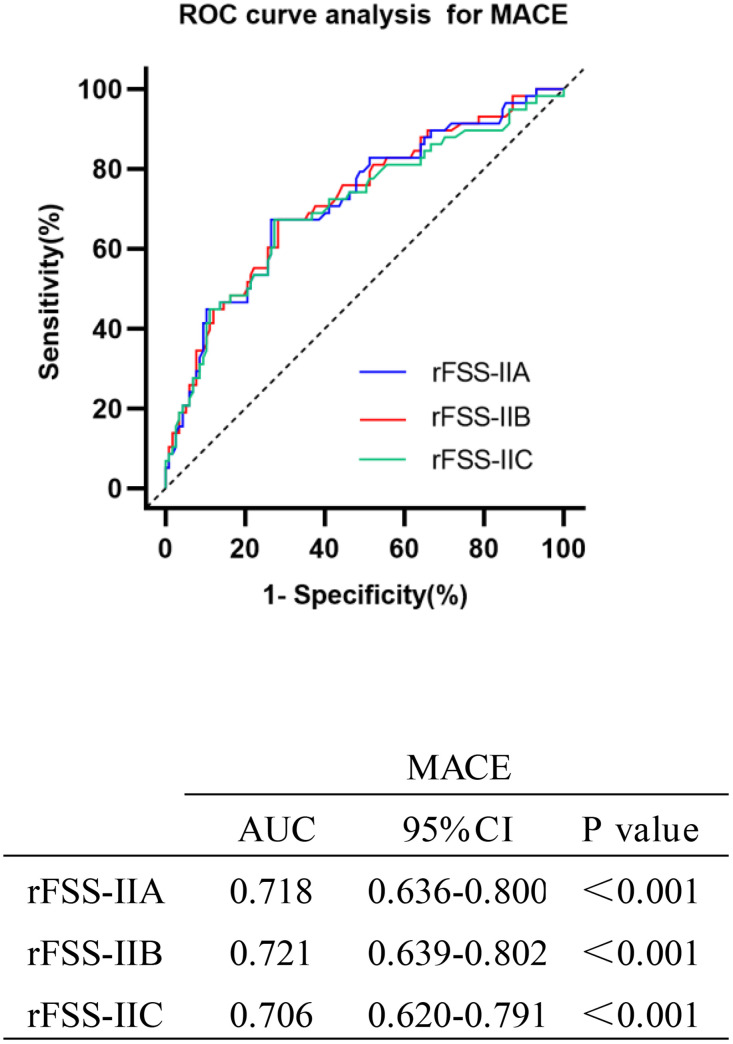
The ROC curves of the three rFSS-II models (A/B/C) as the marker to predict MACE in patients after PCI. The AUCs of tree rFSS-II models (A/B/C) for predicting the occurrence of MACE in patients within 3 years after PCI were shown in the table. MACE, major adverse cardiac events; rFSS-II, residual functional SYNTAX score II; ROC, receiver operating characteristic; PCI, percutaneous coronary intervention; AUC, area under the ROC curve.

Based on the ROC analysis of MACEs (Fig 3), all three rFSS-II models had a cut-off value of 26.2 (rFSS-IIA sensitivity: 67.2%, specificity: 71.8%; rFSS-IIB sensitivity: 67.2%, specificity: 73.5%; rFSS-IIC sensitivity: 67.2%, specificity: 72.4%). Patients were then categorized into low (≤26.2) and high (>26.2) rFSS-II groups. The baseline characteristics of the low (n = 105) and high (n = 70) rFSS-II groups are compared in Table 2, showing significant differences in age, sex, eGFR, LVEF, body mass index (BMI), and smoking status between the groups. Regarding angiographic baseline and treatments, the results did not show any significant differences in the number of residual vessel diseases, location and degree of culprit vessel stenosis, and proportion of drug balloons or drug-eluting stents used.

**Table 2:** Baseline characteristics of the patients stratifed by the rFSS-II.

Per-patient analysis	All subjects(n = 175)	Low(n = 105)	High(n = 70)	P value
Age, years	61.54 ± 12.23	55.27 ± 9.00	70.94 ± 10.27	<.001
Female	141(80.6)	4(3.8)	30(42.9)	<.001
Hypertension	91(52.0)	50(47.6)	41(58.6)	0.155
Smoking	85(48.6)	61(58.1)	24(34.3)	0.002
Hyperlipidemia	82(46.9)	52(49.5)	30(42.9)	0.387
Diabetes	53(30.3)	27(25.7)	26(37.1)	0.107
STEMI	157(89.7)	93(88.6)	64(91.4)	0.542
Previous PCI	10(5.7)	8(7.6)	2(2.9)	0.319
COPD	5(2.9)	1(1.0)	4(5.7)	0.165
PAD	2(1.1)	0(0.0)	2(2.9)	0.159
eGFR, mL/min/1.73m2	78.95 ± 31.07	95.25 ± 26.80	54.50 ± 18.50	<.001
LVEF, %	51.27 ± 9.65	53.36 ± 9.02	48.14 ± 9.79	<.001
BMI, kg/m2	24.18 ± 3.11	24.88 ± 3.15	23.13 ± 2.75	<.001
Angiographic disease severity				0.052
1-vessel disease	50(28.6)	30(28.6)	20(28.6)	
2-vessel disease	54(30.9)	39(37.1)	15(21.4)	
3-vessel disease	71(40.6)	36(34.3)	35(50.0)	
Residual functional severity				0.244
Insignificant stenosis	77(44.0)	50(47.6)	27(38.6)	
1-vessel disease	67(38.3)	41(39.0)	26(37.1)	
2-vessel disease	29(16.6)	13(12.4)	16(22.9)	
3-vessel disease	2(1.1)	1(1.0)	1(1.4)	
Culprit vessel				0.372
LAD	83(47.4)	51(48.6)	32(45.7)	
LCX	21(12.0)	15(14.3)	6(8.6)	
RCA	71(40.6)	39(37.1)	32(45.7)	
Severity of the culprit vessel				0.174
Severe Stenosis	36(20.6)	26(24.8)	10(14.3)	
Subtotal occlusion	13(7.4)	6(5.7)	7(10.0)	
Total occlusion	126(72.0)	73(69.5)	53(75.7)	
DCB	9(5.1)	5(4.8)	4(5.7)	>.999
DES	156(90.2)	95(92.2)	61(87.1)	0.270
rSS	3.00(1.00,7.00)	2.00(1.00,5.00)	5.00(2.00,9.00)	<.001
rFSS	0.00(0.00,3.00)	0.00(0.00,2.00)	2.00(0.00,7.00)	<.001
rSS-II	24.10(18.50,33.10)	20.10(15.85,23.30)	36.15(30.40,41.00)	<.001
rFSS-II	23.80(17.60,32.10)	19.60(15.45,22.60)	35.35(30.20,40.33)	<.001

Data are presented as mean±SD, median (IQR) or n (%). MACEs, major adverse cardiac events; STEMI, ST-elevation myocardial infarction; PCI, percutaneous coronary intervention; COPD, chronic obstructive pulmonary disease; PAD, peripheral arterial disease; eGFR, estimated glomerular filtration rate; LVEF, left ventricular ejection fraction; BMI, body mass index; LAD, left anterior descending; LCX, left circumflex; RCA, right coronary artery; DCB, drug-coated balloon; DES, drug-eluting stents; rSS, residual SYNTAX score; rFSS, residual functional SYNTAX score; rSS-II, residual SYNTAX score II; rFSS-II, residual functional SYNTAX score II.

### Clinical outcomes

**Fig 4:**
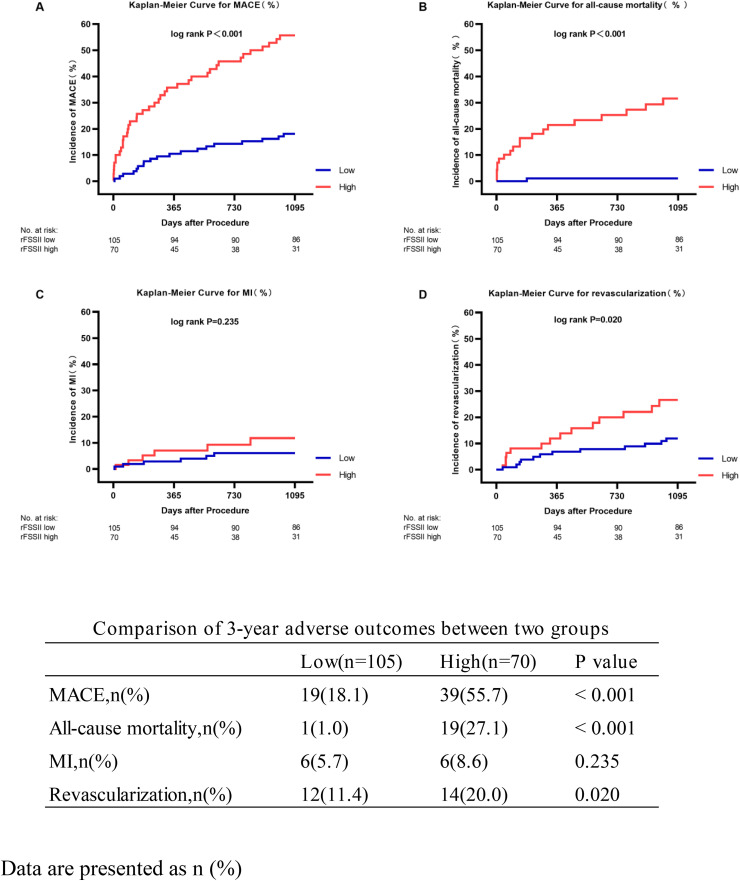
Kaplan-Meier Curves Showing Event Rates Stratified by the rFSS-II Through 3 Years. The groups were stratified by the optimal cutoff value of the rFSS-II determined by receiver operating characteristic curve analysis. (A) MACE, major adverse cardiac events. (B) All-cause mortality. (C) MI, myocardial infarction. (D) Any ischemia-driven revascularization.

Kaplan–Meier analysis revealed that rFSS-II was significantly associated with MACEs, all-cause mortality, and revascularization at 3 years (Fig 4). The low rFSS-II group had a significantly lower incidence of MACEs than the high rFSS-II group. Additionally, differences were observed in all-cause mortality and ischemia-driven revascularization rates between the groups. There were no significant differences in the incidence of MI between the groups.

**Fig 5:**
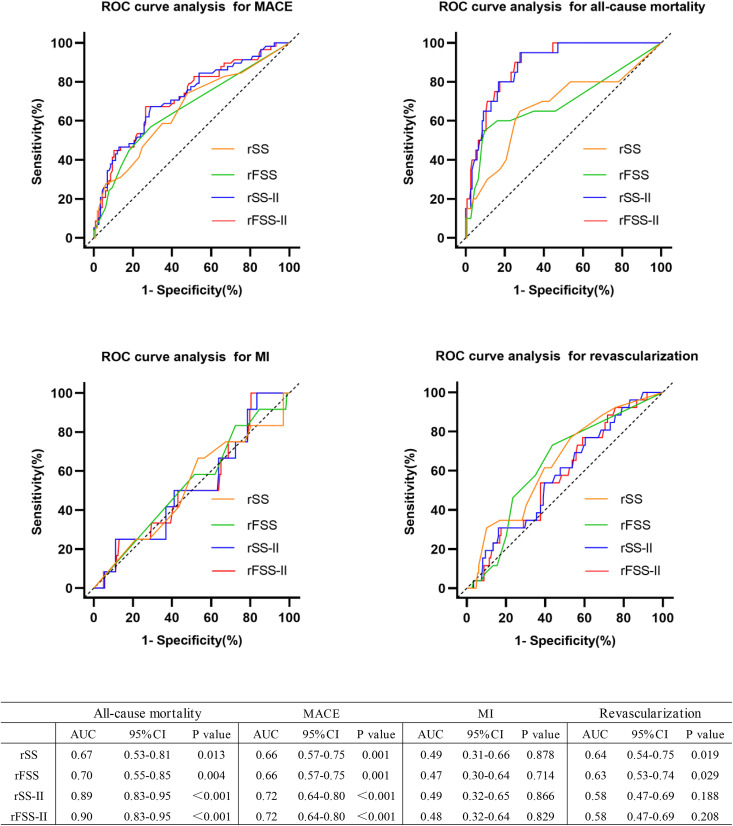
ROC curve analysis of the rFSS-II for adverse outcomes. The AUC of the rFSS-II for predicting the occurrence of adverse outcomes in patients within 3 years after PCI were shown in the table. For all-cause mortality, the p values obtained via DeLong’s test of rFSS-II compared with rSS, rFSS and rSS-II were 0.002, 0.007 and 0.280, respectively. For other outcomes, the P-values obtained by DeLong’s test for rFSS-II and other scores exceeded 0.05.

MACE, major adverse cardiac events; MI, myocardial infarction; PCI, percutaneous coronary intervention; rSS, residual SYNTAX score; rFSS, residual functional SYNTAX score; rSS-II, residual SYNTAX score II; rFSS-II, residual functional SYNTAX score II; ROC, receiver operating characteristic; AUC, area under the ROC curve.

Overall, the area under the ROC curves (AUCs) of the three rFSS-II models were similar, with Model B achieving the highest AUC for predicting MACEs (Fig 3) and all-cause mortality (S1 Fig.). Among the four kinds of scores in Fig 5, the rFSS-II model differed slightly from rSS-II in accuracy; however, the rFSS-II model was more accurate than rFSS and rSS. Regarding predicting revascularization, rSS exhibited higher accuracy than rFSS, while no significant difference was observed between rSS-II and rFSS-II.

After excluding the interference of factors such as hypertension, diabetes, and smoking, multivariable Cox analysis revealed a statistically significant effect of rFSS-II on the occurrence of MACEs (Table 3).

**Table 3:** Independent predictors of 3-year MACE.

Characteristic	Adjusted HR(95% CI)	P value
rFSS-II, per 1 unit	1.08(1.05-1.11)	<.001
Hypertension	1.12(0.67-1.89)	0.668
Smoking	1.71(0.96-3.04)	0.066
Hyperlipidemia	0.61(0.36-1.04)	0.071
Diabetes	1.52(0.89-2.61)	0.128
Previous PCI	1.68(0.59-4.75)	0.332

MACE: major adverse cardiac events; HR, hazard ratio; CI, confidence interval; rFSS-II, residual functional SYNTAX score II; PCI, percutaneous coronary intervention.

### Power of the cohort study

To determine whether our sample size was sufficient to detect a reliable effect, we conducted a power analysis for this cohort study. We used the following formula for calculation.With a fixed sample size of n = 175 and a two-sided alpha level of 0.05, the calculated statistical power (1-β) was 0.99 for detecting the observed effect size. This analysis indicates that the current sample size (n = 175) is adequate to identify an effect of clinical significance.

$\text{n}={(\frac{\text{2}{\text{u}}_{\text{1}-\alpha /\text{2}}\sqrt{\overset{―}{\text{p}}\overset{―}{\text{q}}}\;+{\text{u}}_{\beta }\sqrt{{\text{p}}_{\text{0}}{\text{q}}_{\text{0}}+{\text{p}}_{\text{1}}{\text{q}}_{\text{1}}}}{[{\text{p}}_{\text{1}}-{\text{p}}_{\text{0}}]})}^{\text{2}}$

### Sensitivity analysis of clinical outcomes

To further validate the robustness of our findings, we conducted additional sensitivity analyses using proxy variables for key potential confounders. Medication adherence was approximated using medication renewal records from outpatient follow-ups. Socioeconomic status (SES) was proxied by insurance type, with patients enrolled in rural insurance or no insurance at all classified as the low SES group, and those with employee medical insurance or commercial health insurance classified as the high SES group. Consistency of post-discharge medication therapy was assessed based on discharge prescriptions, with baseline comparisons provided in S3 Table. After adjusting for baseline covariates including hypertension and diabetes, these three proxy variables were simultaneously incorporated into the multivariable Cox regression model. The results demonstrated that the rFSS-II score remained an independent predictor of MACE (adjusted HR per unit: 1.09, 95% CI: 1.06–1.12, P < 0.001), with only marginal changes in the effect size, as detailed in Table 4.

**Table 4:** Cox regression analysis including rroxy variables.

Characteristic	Univariate analysis		Multivariable Analysis	
	HR(95% CI)	P value	HR (95% CI)	P value
rFSS-II, per 1 unit	1.07 (1.04 ~ 1.09)	<.001	1.09 (1.06 ~ 1.12)	<.001
High SES	0.37 (0.19 ~ 0.72)	0.003	0.23 (0.11 ~ 0.47)	<.001
Medication adherence	0.21 (0.11 ~ 0.40)	<.001	0.19 (0.10 ~ 0.38)	<.001
Beta-blocker	1.22 (0.44 ~ 3.37)	0.702	1.39 (0.45 ~ 4.29)	0.568
ACEI/ARB/ARNI	1.07 (0.46 ~ 2.49)	0.874	1.61 (0.65 ~ 3.97)	0.301
CCB	0.78 (0.37 ~ 1.65)	0.523	1.50 (0.66 ~ 3.42)	0.337
Ezetimibe	0.69 (0.29 ~ 1.60)	0.382	1.58 (0.60 ~ 4.18)	0.360

All patients were continued on dual antiplatelet therapy and statin treatment after discharge. HR, hazard ratio; CI, confidence interval; rFSS-II, residual functional SYNTAX score II; SES, socioeconomic status; ACEI, angiotensin-converting enzyme inhibitors; ARB, angiotensin II receptor blocker; ARNI, angiotensin receptor-neprilysin inhibitors; CCB, calcium channel blocker.

## Discussion

In this study, we investigated the prognostic significance of rFSS-II, yielding the following key findings. First, rFSS-II is a novel scoring tool that combines rFSS and clinical factors and has emerged as an independent predictor of MACEs at 3 years post-PCI in patients with AMI. Second, rFSS-II demonstrated superior predictive capability for the all-cause mortality of MACEs compared with other traditional risk-scoring systems except for rSS-II. Third, the predictive accuracy of the three rFSS-II models was similar, and summing the scores of all vessel segments with QFR ≤ 0.8 may offer greater precision.

SS was introduced in 2005 to evaluate lesion complexity and progression in patients [17]. However, it lacks the inclusion of clinical factors, such as age and renal function, recognized as important indicators affecting long-term prognosis post-revascularization. Consequently, it may not accurately predict lesion improvement and prognosis post-PCI [18]. To address this limitation, SS-II was developed; it combines coronary anatomical features and patient clinical factors to evaluate and compare long-term mortality between PCI and CABG treatment strategies [3]. Subsequent adjustments by Bortnick et al. [4] that included additional variables not originally included in SS-II did not change the magnitude of the association, highlighting the importance of incorporating these clinical factors into the scoring system. However, there are significant anatomical differences between post-intervention and untreated vessels, and rSS can effectively reflect patients’ residual diseases and predict cardiovascular events post-PCI [19]. Previous studies have attempted to enhance the predictive ability of rSS by integrating functional parameters [14,20]. Although Rui et al. [21] demonstrated that including clinical factors enhances risk stratification based on rFSS, no study has effectively quantified this risk [21]. Therefore, we aimed to establish a novel scoring system that comprehensively evaluates and quantifies individual patients by considering residual anatomical, functional, and clinical factors.

This study demonstrates that rFSS-II, derived from combining rFSS and clinical factors, aids in the early identification of high-risk patients prone to long-term adverse events among patients with AMI post-PCI. By analyzing the ROC curve, the optimal cut-off value for rFSS-II was, for the first time, determined to be 26.2, leading to patients being categorized into the high and low rFSS-II groups. It was found that the high rFSS-II group exhibited a significantly higher risk of developing MACEs, all-cause mortality, and revascularization than the low rFSS-II group. Furthermore, the related clinical factors (e.g., age, sex, glomerular filtration rate, LVEF, and BMI) involved in calculating the rFSS-II baseline characteristics differed between the groups. The lack of significant differences in COPD and peripheral vascular disease may be attributed to the small sample size.

Functional complete revascularization is an ideal objective of PCI, aiding in assessing patient prognosis and incorporating clinical factors that offer advantages in predicting the clinical outcomes post-PCI [8,21]. A study by Spitaleri et al. [22] demonstrated the feasibility of calculating QFR for non-culprit lesions in STEMI settings. They found that functional incomplete revascularization, as determined by QFR, correlated with poor long-term clinical outcomes, consistent with our results (S1 Table.). The present study is the first to quantify the risks associated with AMI in patients. In the multivariable regression analysis, all variables involved in calculating rFSS-II were excluded to prevent introducing mediating and confounding variables (BMI, COPD, PAD, eGFR, LVEF, LM, age, and sex), thus enhancing result accuracy,and the results of the Cox regression analysis between these clinical factors (incorporated into the rFSS-II model) and the clinical outcome were presented in S2 Table. The results revealed that rFSS-II was an independent predictor of MACEs. For every 1-point increase in rFSS-II, the risk of MACEs during the 3-year follow-up period increased by 8%. Additionally, combining rFSS with clinical factors significantly improved predictive efficiency compared with combining rFSS with functional parameters alone. Notably, the predictive accuracy of the rSS for revascularization underperformed compared to other derived scores, consistent with previous findings [23,24]. This suggests that the progression of residual coronary artery lesions may be the primary factor driving repeat revascularization. Furthermore, we hypothesize that although integrated clinical-anatomical-functional models such as rFSS-II improve the prediction of hard endpoints, they may reduce sensitivity to revascularization. When predicting anatomically driven events, models focusing on vascular characteristics (e.g., rSS/rFSS) demonstrate greater advantage, whereas rFSS-II, which incorporates clinical variables, is more suitable for predicting systemic risk events.

The results of the comparison of the predictive performance of rFSS-II with other scores reveals significant implications. In essence, rFSS-II integrates functional information on the basis of rSS-II, and determines the final vessels to be included in the calculation after secondary screening of the diseased vessels. This process involves double consideration of the morphological and functional characteristics of the vascular lesions. These two scores include different vessels when the lesions are borderline lesions; however, the lesions account for only a small percentage of the total, and their impact on the final score is relatively limited. Therefore, for all patients with MI, the predictive performance of the new score is not significantly improved, but patients with borderline lesions may still be potential beneficiaries. However, after the addition of important clinical factors, the prediction performance has been significantly improved, highlighting that when evaluating coronary artery disease, the anatomical and functional characteristics of the disease, and the general health status of the patient, which may be as important or even more critical for the prognosis of the disease as individualized interventional therapy for coronary artery disease, should be considered.

When discussing the functional significance of rFSS, different studies have employed varying calculation methods and tended to use a combination of anatomy and functional significance as criteria for selecting vessels; thus, there is no universally recognized standard [7,21]. To avoid significant discrepancies in study results due to these differences, we conducted further analysis to assess the accuracy of rFSS values obtained using different calculation methods in predicting clinical outcomes. Notably, guidance from QFR can prevent unnecessary stent placement in lesions with favorable outlooks and detect non-obstructive functional ischemic lesions with negative prognostic consequences [9]. However, Model A primarily assesses anatomic residual stenosis. Thus, opting for Model A over Model B in real-world PCI interventions may overlook non-obstructive functional ischemic lesions. This oversight can lead to an increased risk of adverse events, including non-procedural MI and recurrent angina, necessitating unplanned revascularization procedures [25]. Furthermore, Model B has a larger AUC than the other two calculation models. This suggests that functionally ischemic vessels (QFR ≤ 0.80) may affect patient prognosis regardless of anatomical stenosis. The utilization of Model B may play a more comprehensive role in guiding surgical treatment. In designing Model C, we primarily focused on the following aspect: in cases of tandem or diffuse lesions, even if the QFR value of the entire vessel is ≤ 0.80, only a single stenosis or segmental diffuse lesion may not result in functional ischemia in the whole vessel. Functional ischemia can occur only when the functional changes of various vessels are combined. Therefore, we assigned a higher weight to the proximal stenotic vessel in our calculations. Our study indicated that the accuracy of Model C might be lower than that of other models. Considering that the proportion of such vessels may be small, this calculation method may not apply to all vessels, especially in cases of severe multi-segment functional ischemia where the obtained scores may be low. It is worth mentioning that there is no significant difference between the three models and between rFSS-II and rSS-II in predicting MACEs, mainly due to the small proportion of functional parameters in rFSS-II. However, the addition of functional parameters can increase the accuracy of prediction results to a certain extent compared with other traditional scores except rSS-II. The differing discriminative abilities of the three models still imply that prioritizing the restoration of the physiological function of all vessels without increasing surgical complications could be a worthwhile PCI strategy.

We observed a significant discrepancy in the incidence of MACE during hospitalization and after discharge. During the index hospitalization, only six out of 175 patients (3.4%) experienced MACE. However, during an extended follow-up period of 3 years post-discharge, the incidence of MACE increased to 58 out of 175 patients (33.1%). Upon further investigation, it was noted that 46 of these patients (79.3%) opted for follow-up treatment at a primary hospital or clinic closer to their residences rather than returning to our institution. Among this cohort, a substantial proportion (35 individuals, 76.1%) were rural residents. It is hypothesized that the lower adherence to post-discharge treatment regimens observed in this demographic may contribute to the elevated MACE rates noted in our follow-up analysis. This observation may indicate the importance of considering geographical and socio-economic factors in assessing and managing cardiovascular risk post-hospitalization.Sensitivity analysis with proxy variables confirmed the robustness of our primary result.

Our findings are particularly significant in the high-risk context of AMI. A recent multicenter study on the assessment of non-culprit lesions in STEMI patients confirmed that QFR maintains strong diagnostic value even in the acute phase, demonstrating the feasibility of translating QFR-based functional information into a long-term prognostic prediction model [26]. This population closely overlaps with ours and strongly supports the considerable potential of rFSS-II for use in AMI patients. These results carry direct clinical implications for guiding risk stratification and intensity of long-term follow-up in AMI patients after discharge.

Furthermore, the rFSS-II score examined in this study shows significant convergence with recent findings from the team of Dou Kefei based on the FAVOR III China trial [27]. Their study identified eGFR, age, LVEF, BMI, SS/rSS, stent parameters, LCX lesions, and QFR gray-zone values as independent risk factors predicting 2-year MACE. It is noteworthy that these factors correspond to clinical, anatomical, and functional domains, respectively. The rFSS-II system effectively addresses this complexity by integrating such multidimensional information with clinical characteristics into a comprehensive decision-support tool. Therefore, our study methodologically corroborates the clinical observations made by Dou Kefei’s team and offers a practical solution that consolidates multiple scattered risk factors into a single, quantifiable risk score.

Our study reinforces that the rFSS-II scoring system, integrating anatomical, functional, and clinical factors, can better identify patients at high risk of AMI post-PCI. Patients should be stratified based on their risks to enhance their clinical prognosis and administered individualized comprehensive treatment early.

### Study limitations

Our study has several limitations. First, it is a single-center cohort clinical study with a small sample size, indicating inherent selection bias. Future studies should include a larger sample size to validate our findings. Second, the requirement of only one projection for functional tests using AngioPlus Galley 2.0 may fail to distinguish a small number of eccentric lesions, potentially affecting the vessel selection for calculations. Third, due to the small proportion of functionology in this score, models A–C showed a small difference in predicting late outcomes; therefore, more studies are needed to confirm it.

## Conclusions

rFSS, when combined with clinical factors, showed a good predictive value in predicting 3-year MACEs. Moreover, calculating the total score of all vascular segments with QFR ≤ 0.80 may provide enhanced predictive capability.

### Glossary

SYNTAX: Synergy Between Percutaneous Coronary Intervention with Taxus and Cardiac Surgery; a scoring system used to assess the complexity of coronary artery disease.
rFSS: Residual Functional SYNTAX Score; a measure used to evaluate the remaining complexity of coronary artery disease after intervention.
PCI: Percutaneous Coronary Intervention; a non-surgical procedure used to treat narrowing of the coronary arteries of the heart.
QFR: Quantitative Flow Ratio; a measurement used to assess the severity of coronary artery stenosis using digital subtraction angiography.
MACE: Major Adverse Cardiac Events; a term used to describe serious cardiovascular events, including death, myocardial infarction, and stroke.
AMI: Acute Myocardial Infarction; commonly known as a heart attack, it is a condition where blood flow to a part of the heart is blocked, causing damage to the heart muscle.
CI: Confidence Interval; a statistical range that is likely to contain the true value of a measurement or estimate.
ROC: Receiver Operating Characteristic; a graphical plot used to assess the performance of a diagnostic test or model.
HR: Hazard Ratio; a measure used in survival analysis to compare the risk of a particular event occurring between two groups.
S-II: SYNTAX Score II; an updated version of the SYNTAX score that incorporates additional clinical variables to better assess patient risk.
COPD: Chronic Obstructive Pulmonary Disease; a progressive lung disease characterized by long-term breathing problems and poor airflow.
PAD: Peripheral Arterial Disease; a condition in which narrowed arteries reduce blood flow to the limbs.
eGFR: Estimated Glomerular Filtration Rate; a measure of kidney function that estimates how well the kidneys are filtering blood.
LVEF: Left Ventricular Ejection Fraction; a measurement of the percentage of blood that is pumped out of the left ventricle with each contraction.
LM: Left Main (Disease); a condition involving the left main coronary artery, which supplies blood to a large portion of the heart.
rSS: Residual SYNTAX Score; a score indicating the remaining complexity of coronary artery disease after treatment.
FFR: Fractional Flow Reserve; a diagnostic technique used to measure blood flow and assess the significance of coronary artery stenosis.
CABG: Coronary Artery Bypass Grafting; a surgical procedure used to improve blood flow to the heart by diverting blood around blocked or narrowed coronary arteries.
DSA: Digital Subtraction Angiography; an imaging technique used to visualize vessels and organs by subtracting pre-contrast images from post-contrast images.
STEMI: ST-Elevation Myocardial Infarction; a type of heart attack characterized by a significant elevation in the ST segment of an electrocardiogram (ECG).
BMI: Body Mass Index; a measure of body fat based on height and weight.
AUC: Area Under the Curve; a statistical measure used to evaluate the performance of a diagnostic test, representing the probability that the test will correctly distinguish between positive and negative cases.
MI: Myocardial Infarction; a medical term for a heart attack, where the blood flow to part of the heart is blocked, causing damage to the heart muscle.

## Supporting information

S1 Fig ROC curves analysis of the tree rFSS-II models (A/B/C) for adverse outcomes. The AUCs of the tree rFSS-II models (A/B/C) for predicting the occurrence of adverse outcomes in patients within 3 years after PCI were shown in the table. MI, myocardial infarction; rFSS-II, residual functional SYNTAX score II; PCI, percutaneous coronary intervention; ROC, receiver operating characteristic; AUC, area under the ROC curve.
(DOCX)

S1 Table Comparison of outcomes according to functional revascularization. Data are presented as n (%). FCR, functional complete revascularization; FIR, functional incomplete revascularization; MACE, major adverse cardiac events; MI, myocardial infarction.
(DOCX)

S2 Table Cox Analysis of Clinical Factors in the rFSS-II. COPD, chronic obstructive pulmonary disease; PAD, peripheral arterial disease; LVEF, left ventricular ejection fraction; eGFR, estimated glomerular filtration rate; LM,unprotected left main disease.
(DOCX)

S3 Table Characteristics of Proxy Variables Stratified by rFSS-II. rFSS-II, residual functional SYNTAX score II; SES, socioeconomic status; ACEI, angiotensin-converting enzyme inhibitors; ARB, angiotensin II receptor blocker; ARNI, angiotensin receptor-neprilysin inhibitors; CCB, calcium channel blocker. (DOCX)

## References

[1] Keeley EC, Boura JA, Grines CL. Primary angioplasty versus intravenous thrombolytic therapy for acute myocardial infarction: a quantitative review of 23 randomised trials. Lancet. 2003;361(9351):13–20. doi: 10.1016/S0140-6736(03)12113-7 12517460

[2] Généreux P, Palmerini T, Caixeta A, Rosner G, Green P, Dressler O, et al. Quantification and impact of untreated coronary artery disease after percutaneous coronary intervention: the residual SYNTAX (Synergy Between PCI with Taxus and Cardiac Surgery) score. J Am Coll Cardiol. 2012;59(24):2165–74. doi: 10.1016/j.jacc.2012.03.010 22483327 PMC3725642

[3] Farooq V, van Klaveren D, Steyerberg EW, Meliga E, Vergouwe Y, Chieffo A, et al. Anatomical and clinical characteristics to guide decision making between coronary artery bypass surgery and percutaneous coronary intervention for individual patients: development and validation of SYNTAX score II. Lancet. 2013;381(9867):639–50. doi: 10.1016/S0140-6736(13)60108-7 23439103

[4] Bortnick AE, Shitole SG, Hashim H, Khullar P, Park M, Weinreich M, et al. Residual SYNTAX II score and long-term outcomes post-ST-elevation myocardial infarction in an urban US cohort: the Montefiore STEMI registry. Coron Artery Dis. 2022;33(3):206–12. doi: 10.1097/MCA.0000000000001074 34049323 PMC8617039

[5] Nakamura M, Yamagishi M, Ueno T, Hara K, Ishiwata S, Itoh T, et al. Prevalence of visual-functional mismatch regarding coronary artery stenosis in the CVIT-DEFER registry. Cardiovasc Interv Ther. 2014;29(4):300–8. doi: 10.1007/s12928-014-0259-3 24664513

[6] Park S-J, Kang S-J, Ahn J-M, Shim EB, Kim Y-T, Yun S-C, et al. Visual-functional mismatch between coronary angiography and fractional flow reserve. JACC Cardiovasc Interv. 2012;5(10):1029–36. doi: 10.1016/j.jcin.2012.07.007 23078732

[7] Choi KH, Lee JM, Koo BK, Nam CW, Shin ES, Doh JH, et al. Prognostic implication of functional incomplete revascularization and residual functional SYNTAX score in patients with coronary artery disease. JACC Cardiovasc Interv. 2018;11(3):237–45. 29361444 10.1016/j.jcin.2017.09.009

[8] Kobayashi Y, Nam C-W, Tonino PAL, Kimura T, De Bruyne B, Pijls NHJ, et al. The prognostic value of residual coronary stenoses after functionally complete revascularization. J Am Coll Cardiol. 2016;67(14):1701–11. doi: 10.1016/j.jacc.2016.01.056 27056776

[9] Song L, Tu S, Sun Z, Wang Y, Ding D, Guan C, et al. Quantitative flow ratio-guided strategy versus angiography-guided strategy for percutaneous coronary intervention: Rationale and design of the FAVOR III China trial. Am Heart J. 2020;223:72–80. doi: 10.1016/j.ahj.2020.02.015 32179258

[10] Tu S, Barbato E, Köszegi Z, Yang J, Sun Z, Holm NR, et al. Fractional flow reserve calculation from 3-dimensional quantitative coronary angiography and TIMI frame count: a fast computer model to quantify the functional significance of moderately obstructed coronary arteries. JACC Cardiovasc Interv. 2014;7(7):768–77. doi: 10.1016/j.jcin.2014.03.004 25060020

[11] Tu S, Westra J, Yang J, von Birgelen C, Ferrara A, Pellicano M, et al. Diagnostic accuracy of fast computational approaches to derive fractional flow reserve from diagnostic coronary angiography: the international multicenter FAVOR pilot study. JACC Cardiovasc Interv. 2016;9(19):2024–35. 27712739 10.1016/j.jcin.2016.07.013

[12] Xu B, Tu S, Qiao S, Qu X, Chen Y, Yang J, et al. Diagnostic accuracy of angiography-based quantitative flow ratio measurements for online assessment of coronary stenosis. J Am Coll Cardiol. 2017;70(25):3077–87. 29101020 10.1016/j.jacc.2017.10.035

[13] Westra J, Andersen BK, Campo G, Matsuo H, Koltowski L, Eftekhari A, et al. Diagnostic performance of in-procedure angiography-derived quantitative flow reserve compared to pressure-derived fractional flow reserve: the FAVOR II Europe-Japan study. J Am Heart Assoc. 2018;7(14):e009603. doi: 10.1161/JAHA.118.009603 29980523 PMC6064860

[14] Tang J, Lai Y, Tu S, Chen F, Yao Y, Ye Z, et al. Quantitative flow ratio-guided residual functional SYNTAX score for risk assessment in patients with ST-segment elevation myocardial infarction undergoing percutaneous coronary intervention. EuroIntervention. 2021;17(4):e287–93. doi: 10.4244/EIJ-D-19-00369 31589145 PMC9724850

[15] Thygesen K, Alpert JS, Jaffe AS, Chaitman BR, Bax JJ, Morrow DA, et al. Fourth universal definition of myocardial infarction (2018). J Am Coll Cardiol. 2018;72(18):2231–64. 30153967 10.1016/j.jacc.2018.08.1038

[16] Mancini GBJ, Hartigan PM, Shaw LJ, Berman DS, Hayes SW, Bates ER, et al. Predicting outcome in the COURAGE trial (Clinical Outcomes Utilizing Revascularization and Aggressive Drug Evaluation): coronary anatomy versus ischemia. JACC Cardiovasc Interv. 2014;7(2):195–201. doi: 10.1016/j.jcin.2013.10.017 24440015

[17] Sianos G, Morel M-A, Kappetein AP, Morice M-C, Colombo A, Dawkins K, et al. The SYNTAX Score: an angiographic tool grading the complexity of coronary artery disease. EuroIntervention. 2005;1(2):219–27. 19758907

[18] Collet C, Onuma Y, Andreini D, Sonck J, Pompilio G, Mushtaq S, et al. Coronary computed tomography angiography for heart team decision-making in multivessel coronary artery disease. Eur Heart J. 2018;39(41):3689–98. doi: 10.1093/eurheartj/ehy581 30312411 PMC6241466

[19] Song Y, Gao Z, Tang X, Jiang P, Xu J, Yao Y, et al. Impact of residual SYNTAX score on clinical outcomes after incomplete revascularisation percutaneous coronary intervention: a large single-centre study. EuroIntervention. 2017;13(10):1185–93. doi: 10.4244/EIJ-D-17-00132 28760723

[20] Lee SH, Choi KH, Lee JM, Shin D, Hwang D, Kim HK, et al. Residual functional SYNTAX score by quantitative flow ratio and improvement of exercise capacity after revascularization. Catheter Cardiovasc Interv. 2021;97(4):E454–66. doi: 10.1002/ccd.29118 32618423

[21] Zhang R, Wang H-Y, Dou K, Yin D, Zhu C, Feng L, et al. Outcomes of functionally complete vs incomplete revascularization: insights from the FAVOR III china trial. JACC Cardiovasc Interv. 2022;15(24):2490–502. doi: 10.1016/j.jcin.2022.10.014 36543443

[22] Spitaleri G, Tebaldi M, Biscaglia S, Westra J, Brugaletta S, Erriquez A, et al. Quantitative flow ratio identifies nonculprit coronary lesions requiring revascularization in patients with ST-segment-elevation myocardial infarction and multivessel disease. Circ Cardiovasc Interv. 2018;11(2):e006023. doi: 10.1161/CIRCINTERVENTIONS.117.006023 29449325

[23] Song Y, Gao Z, Tang X-F, Jiang P, Xu J-J, Yao Y, et al. Impact of residual SYNTAX score and its derived indexes on clinical outcomes after percutaneous coronary intervention: data from a large single center. Chin Med J (Engl). 2018;131(12):1390–6. doi: 10.4103/0366-6999.233958 29893355 PMC6006821

[24] Yan L, Li P, Wang Y, Han D, Li S, Jiang M, et al. The incremental prognostic value of the clinical residual SYNTAX score for patients with chronic renal insufficiency undergoing percutaneous coronary intervention. Front Cardiovasc Med. 2021;8:647720. doi: 10.3389/fcvm.2021.647720 33937361 PMC8082103

[25] Pijls NHJ, van Schaardenburgh P, Manoharan G, Boersma E, Bech J-W, van’t Veer M, et al. Percutaneous coronary intervention of functionally nonsignificant stenosis: 5-year follow-up of the DEFER Study. J Am Coll Cardiol. 2007;49(21):2105–11. doi: 10.1016/j.jacc.2007.01.087 17531660

[26] Alfonso Rodríguez E, Gómez-Lara J, López-Palop R, Gutiérrez E, Goncalves Ramírez LR, Valencia J, et al. Diagnostic accuracy of quantitative flow ratio for nonculprit intermediate lesions in patients with ST-segment elevation myocardial infarction. Rev Esp Cardiol (Engl Ed). 2026;79(1):49–58. doi: 10.1016/j.rec.2025.05.010 40451507

[27] Dou K, Wang H, Guan C, Song L, Wijns W, editors. Clinical and angiographic features of patients likely to have MACE despite online QFR-guided PCI. Paris, France; 2025. https://europcr2025.europa-inviteo.com/gws/data/onglet27/module1/modalPreview.php?langue=fr¶mProjet=64799

