# Supplementary figures and images for: Prognostic significance of residual functional SYNTAX score II in acute myocardial infarction
Source: PLoS One. 2026 Jan 23;21(1):e0340784. doi: 10.1371/journal.pone.0340784 (PMC12829799; doi:10.1371/journal.pone.0340784)

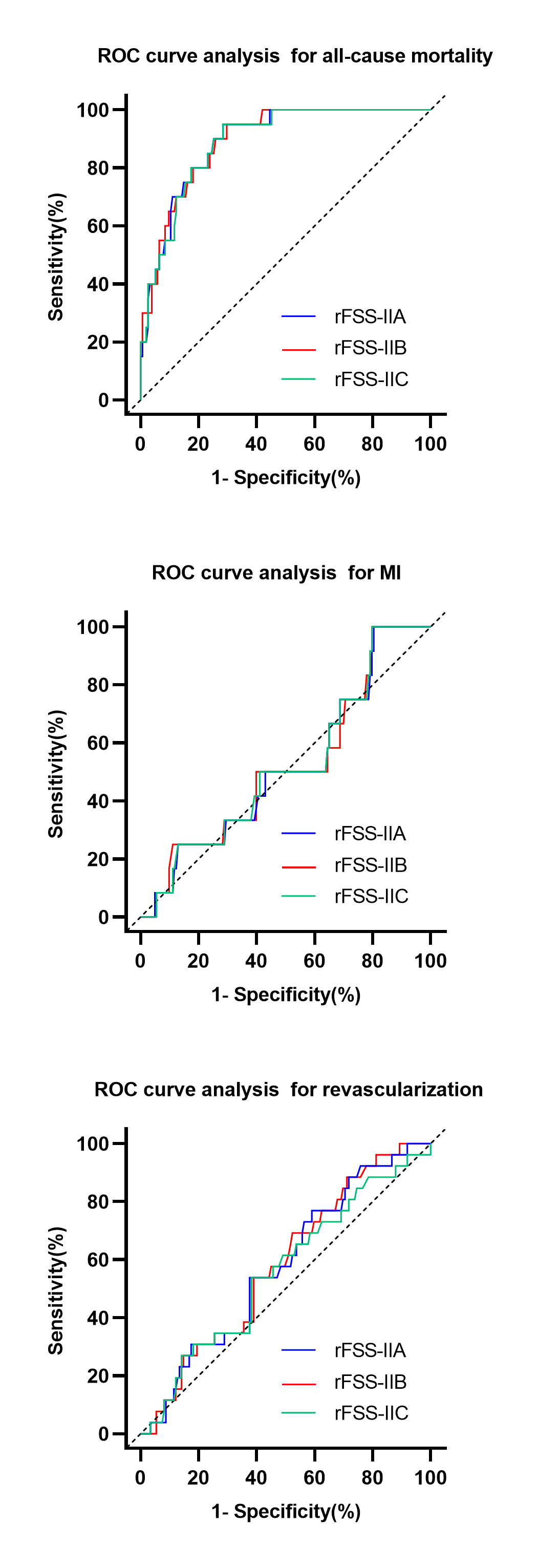


S1 Fig.

Supplement: S1 Fig — The AUCs of the tree rFSS-II models (A/B/C) for predicting the occurrence of adverse outcomes in patients within 3 years after PCI were shown in the table. MI, myocardial infarction; rFSS-II, residual functional SYNTAX score II; PCI, percutaneous coronary intervention; ROC, receiver operating characteristic; AUC, area under the ROC curve. (DOCX) [file pone.0340784.s001.docx]
